# Targeting bacterial pathogenesis by inhibiting virulence-associated Type III and Type IV secretion systems

**DOI:** 10.3389/fcimb.2022.1065561

**Published:** 2023-01-10

**Authors:** Nadja Blasey, Daria Rehrmann, Anna Katharina Riebisch, Sabrina Mühlen

**Affiliations:** Department of Molecular Immunology, Ruhr-University Bochum, Bochum, Germany

**Keywords:** T3SS, T4SS, bacterial pathogenesis, anti-virulence, pathoblocker

## Abstract

Infections caused by Gram-negative pathogens pose a major health burden. Both respiratory and gastrointestinal infections are commonly associated with these pathogens. With the increase in antimicrobial resistance (AMR) over the last decades, bacterial infections may soon become the threat they have been before the discovery of antibiotics. Many Gram-negative pathogens encode virulence-associated Type III and Type IV secretion systems, which they use to inject bacterial effector proteins across bacterial and host cell membranes into the host cell cytosol, where they subvert host cell functions in favor of bacterial replication and survival. These secretion systems are essential for the pathogens to cause disease, and secretion system mutants are commonly avirulent in infection models. Hence, these structures present attractive targets for anti-virulence therapies. Here, we review previously and recently identified inhibitors of virulence-associated bacterial secretions systems and discuss their potential as therapeutics.

## Introduction

The development and spread of antimicrobial resistance (AMR) are among the biggest challenges for global health (Jee et al., 2018; Zhang et al., 2022). With the microbiota serving as a reservoir for resistance genes that can be passed on to pathogens *via* horizontal gene transfer, multi-resistant pathogens emerge in ever greater numbers (Kent et al., 2020; Hofer, 2022). As antibiotics target bacterial growth and survival mechanisms, this leads to an increase in selective pressure which will only aid the development of new resistance mutations (Martinez and Baquero, 2000; Perry and Wright, 2014). Furthermore, there is an increase in the prescription of last-resort antibiotics (Osei Sekyere, 2016; Klein et al., 2018). Their increased use will lead to further spread of resistance and limit treatment options for bacterial infections. In 2019, an estimated 1.2 million deaths globally were associated with antibiotic-resistant or multi-resistant bacterial infections (Antimicrobial Resistance, 2022). For Europe alone, this attributed to a cost of 1.5 billion Euros in healthcare-associated expenses and productivity loss (Anderson et al., 2019). Furthermore, only 17 new antimicrobials have been approved for use in the past decade (Chahine et al., 2022).

Due to the increase in AMR and the speed at which resistances to new antibiotics arise, research has shifted some of its focus to anti-virulence strategies, which interfere with virulence properties such as toxin production, quorum sensing or adhesion, thus preventing pathogenesis. Here, one of the aims is to identify so-called pathoblockers, compounds that target bacterial virulence factors without affecting the growth or survival of either the pathogen or the microbiota. This strategy renders bacteria avirulent but does not kill them, limiting survival pressure. Moreover, avirulent bacteria either cannot colonize or may be recognized and cleared by the immune system (Keyser et al., 2008; Duncan et al., 2012; Mühlen and Dersch, 2016; Hotinger et al., 2021). Furthermore, many virulence factors at which pathoblockers are targeted are exposed on the bacterial cell surface, bypassing the expulsion by bacterial efflux pumps (Smith and Romesberg, 2007).

Bacterial virulence factors include toxins, adhesins, invasins, secretion systems, and translocated proteins (effectors) essential for adherence, colonization, invasion, and disease establishment (Rasko and Sperandio, 2010; Mühlen and Dersch, 2016; Fleitas Martinez et al., 2019). Eleven different bacterial secretion systems have been described to date (Abrusci et al., 2014; Green and Mecsas, 2016; Grossman et al., 2021; Palmer et al., 2021). Type III and Type IV secretion systems (T3SSs and T4SSs, respectively) are commonly found in Gram-negative pathogenic bacteria and are associated with bacterial pathogenesis in eukaryotic hosts (Coburn et al., 2007; Keyser et al., 2008; Baron, 2010). They translocate effector proteins from the bacteria into the host cell cytosol. The genes encoding for T3- and T4SSs are mainly found in pathogenic bacteria and are usually located within mobile genetic elements associated with virulence, such as pathogenicity islands or plasmids. T3SSs are found in more than 25 species of Gram-negative pathogens, including pathogenic *E. coli*, *Salmonella*, *Chlamydia*, and *Pseudomonas* (Cornelis, 2006; Coburn et al., 2007; Cornelis, 2010). T4SSs are associated with virulence in pathogens such as *Legionella*, *Neisseria*, *Helicobacter*, and *Coxiella* (Cascales and Christie, 2003). Virulence-associated T3- and T4- secretion systems can also be found in plant pathogens including *Pseudomonas syringae*, *Agrobacterium tumefaciens* and *Xanthomonas* spp. (Hueck, 1998; Cascales and Christie, 2003; Mansfield et al., 2012). However, the focus of this review will be on human pathogenic bacteria. Both, T3- and T4SSs span the inner and outer bacterial membrane and protrude from the bacterial surface. Upon contact with a host cell, a pore is formed in the host cell membrane at the tip of the secretion system, which creates a connection from the pathogen to the host cell, enabling it to translocate effector proteins directly into the host cell cytosol, where they subvert cell signaling pathways in favor of bacterial survival and persistence (Dean, 2011; Green and Mecsas, 2016; Bienvenu et al., 2021).

**Figure 1:**
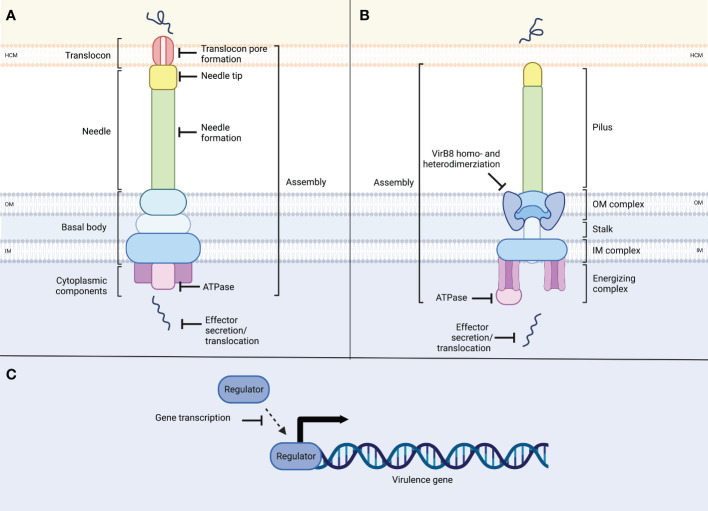
Schematic overview of the structure of type III and type IV secretion systems and potential inhibitor targets. **(A)** T3SSs consist of ring-shaped structures which form the basal body, spanning the inner (IM) and outer membrane (OM) of the bacterium. Cytoplasmic components, including the ATPase complex power needle assembly as well as the translocation of effector proteins. The needle ends in the needle tip, and the translocon forms a pore within the host cell membrane (HCM) through which unfolded effector proteins are translocated into the host cell. T3SSs target sites include secretion system assembly, needle or needle tip formation, translocon pore formation, ATPase function or effector protein secretion/translocation. **(B)** T4SSs consists of ring-like structures in the outer and inner membranes, which are connected through the stalk. At the inner membrane complex, an ATPase complex is located, providing energy for assembly and protein translocation. The pilus connects to the host cell membrane through which unfolded effector proteins are secreted into the host cell. Targets in T4SSs are secretion system assembly, VirB8 dimerization, ATPase function and effector secretion. **(C)** Additionally, inhibition can target virulence regulators, interfering with virulence gene or secretion system transcription.

T3SSs encoded by Gram-negative pathogens play a central role in virulence (Coburn et al., 2007; Cornelis, 2010), making them promising targets for pathoblockers (Keyser et al., 2008; Duncan et al., 2012; Beckham and Roe, 2014; Mühlen and Dersch, 2016; Boudaher and Shaffer, 2019; Hotinger et al., 2021). They are evolutionarily related and therefore highly similar to flagellar T3SSs and consist of more than 20 proteins (Hueck, 1998; Cornelis, 2010; Abby and Rocha, 2012; Deng et al., 2017). T3SSs can be divided into cytosolic components, the basal body, and the needle. The cytosolic components include an ATPase required for unfolding effector proteins and powering their translocation. The cytosolic components are located underneath the basal body, which consists of an inner and an outer ring integrated into the inner and outer bacterial membrane, respectively. The needle complex consists of a base that is integrated into the bacterial membrane and a needle-shaped extension that protrudes from the bacterial cell (**Figure 1A**). In some instances, such as the T3SSs of pathogenic *E. coli*, the needle is followed by a filament, which extends from the former. The needle or filament ends in the tip complex, which is involved in sensing eukaryotic cells. Once in contact with a host cell, the proteins that make up the translocon are translocated through the needle and inserted into the host cell membrane where they form a pore (Dey et al., 2019). As the structures and functions of the secretion systems show high conservation between the different strains of bacteria, so do their synthesis and assembly (Notti and Stebbins, 2016; Condry and Nilles, 2017; Deng et al., 2017; Horna and Ruiz, 2021; Jenkins et al., 2022).

Similar to T3SSs, T4SSs are functionally and structurally diverse (Cascales and Christie, 2003; Fronzes et al., 2009; Bhatty et al., 2013; Low et al., 2014). They are found in Gram-negative and Gram-positive bacteria and can be separated into minimized and expanded T4SSs (Grohmann et al., 2018; Costa et al., 2021; Liu et al., 2022; Sheedlo et al., 2022). They consist of three large groups: bacterial conjugation systems, DNA uptake and release systems, and effector translocators (Fronzes et al., 2009; Wallden et al., 2010; Sgro et al., 2019). The latter subgroup includes the virulence-associated T4SSs used by bacterial pathogens to translocate effector proteins or protein-DNA complexes into host cells to promote their proliferation and survival inside host cells (Cascales and Christie, 2003). The T4SS itself consists of the inner membrane complex spanning the bacterial inner membrane and the outer membrane core complex, which are connected by a stalk. Cytoplasmic components of the inner membrane complex include energy-generating ATPases. A pilus extends from the outer membrane core complex into the extracellular space and enhances protein and nucleic acid transport to the target cells (**Figure 1B**). Several components are conserved among the T4SSs from different pathogens. However, there are also species-specific features, including variations in the number of components and differences in the size and symmetries of the complexes (Low et al., 2014). Thus far, the structures of four T4SS have been identified, including the Vir, Cag, and Dot/Icm T4SS families (Cascales and Christie, 2003; Vincent et al., 2006; Wallden et al., 2010; Sgro et al., 2019; Sheedlo et al., 2022). The Vir secretion system belongs to the minimized T4SSs. First described in *Helicobacter pylori*, it has also been identified in *Brucella* and *Bartonella* (Cascales and Christie, 2003). The Vir T4SS can further be found in the plant pathogen *A. tumefaciens* (Cascales and Christie, 2003). It is composed of 12 subunits, which are VirB1 to VirB11 and VirD4 (Low et al., 2014). A key component of the Vir T4SS is the protein VirB8. To fulfill its function in system assembly, it dimerizes with either VirB8 or VirB10, forming the inner membrane channel of the T4SS together with VirB6 (Paschos et al., 2011; Sivanesan and Baron, 2011). Furthermore, the complex consists of the pilus, which is composed of a major and minor subunit, and the outer membrane complex. Three ATPases (VirB4, VirB11, and VirD4) assist in the assembly of this system (Savvides et al., 2003; Low et al., 2014). Expanded T4SSs are found in the human pathogens *H. pylori, Legionella pneumophila*, and *Coxiella burnetii* (Cascales and Christie, 2003; Nagai and Kubori, 2011). First described in *H. pylori* which translocates the oncogenic effector protein CagA, this family of secretion systems is called the Cag T4SS family (Segal et al., 1998; Fischer, 2011; Cover et al., 2020). The Cag T4SS is a multiprotein complex composed of 20 different proteins. Like the Vir T4SS, it spans the inner and the outer membrane and contains three putative ATPases, essential for CagA secretion (Fischer, 2011; Chung et al., 2019; Hu et al., 2019). The Dot/Icm system of *L. pneumophila* is one of the best-studied T4SSs. It is composed of at least 27 components that assemble into different subcomplexes, including the outer membrane complex, a periplasmic ring, and the inner membrane complex. A channel at the center of the stalk connects the outer and inner membrane complex. Cytosolic ATPases (DotL, DotB, DotO) provide energy for protein translocation through the T4SS (Chetrit et al., 2018; Ghosal et al., 2019).

The virulence-associated T3SS and T4SSs have been at the center of many high-throughput screens in an attempt to identify inhibitors that block the pathogenicity mediated by translocated effector proteins (Baron, 2010; Duncan et al., 2012; Mühlen and Dersch, 2016; Boudaher and Shaffer, 2019; Pendergrass and May, 2019; Hotinger et al., 2021). Many studies used known secretion system-induced virulence phenotypes, such as secretion system-mediated hemolysis, translocation of effector proteins by effector-reporter fusion proteins, effector secretion, or effector-mediated outcomes such as NF-κB induction using reporter gene fusions as the basis of their screens (Beckham and Roe, 2014; Fasciano et al., 2019). Particularly in recent years, there has been a shift toward structure-based computational predictions. Furthermore, several previously identified inhibitors have been modified in attempts to increase their potency, permeability, or target specificity. As the T3SSs of Gram-negative pathogens show very high architectural homology, the aim is to improve inhibitory compounds to identify novel substances with low toxicity, a broad target spectrum, and high selectivity for T3SS-positive pathogens. While many studies have aimed at developing or identifying inhibitors targeting the T3SS of bacteria, fewer have focused on pathogens using T4SSs (Boudaher and Shaffer, 2019). This is unfortunate because bacterial pathogens expressing T4SSs also cause a diverse range of severe diseases. In the case of *H. pylori*, a link to the development of gastric cancer has even been proven (Ishaq and Nunn, 2015; Norwood et al., 2022). Furthermore, as T4SSs were shown to translocate not only effector proteins but also DNA, inhibiting these systems may also aid in reducing the transfer and spread of acquired resistance genes within a community (Juhas et al., 2008; Boudaher and Shaffer, 2019). While no natural inhibitors were found to date that target T4SSs, several chemical compounds have been identified (Boudaher and Shaffer, 2019).

Several processes present themselves as potential targets for anti-virulence strategies: synthesis of needle components, assembly of the secretion system, interaction with the host cell, and secretion/translocation of the substrates (**Figure 1**). Due to the high similarity between each family of secretion systems, inhibitors can likely be found that target not only one but also several different pathogens at once. Furthermore, as only pathogenic bacteria express these secretion systems, non-pathogenic bacteria will not be targeted. Additionally, as secretion system inhibitors are selected for lack of impact on bacterial survival, the selective pressure to develop resistance should remain low.

## Salicylidene acylhydrazides

Salicylidene acylhydrazides (SAHs) are a group of potent chemical inhibitors active against the T3- and T4SSs of several pathogens, including *Salmonella, Pseudomonas*, *Chlamydia*, and *Brucella*. They were among the earliest identified secretion system inhibitors and remain the best-studied compound family to date (**Table 1**) (Duncan et al., 2012; Beckham and Roe, 2014; Mühlen and Dersch, 2020).

**Table 1:** Salicylidene acylhydrazides.

Compound name(s)	Molecular target	Effective against	Reference
INP0007, INP0010, INP0341, INP0400, ME0177, ME0192	Unknown	*Chlamydia*	(Slepenkin et al., 2003; Muschiol et al., 2006; Wolf et al., 2006; Bailey et al., 2007; Slepenkin et al., 2007; Slepenkin et al., 2011; Ur-Rehman et al., 2012; Pedersen et al., 2014)
INP0010, INP0031*, INP0401, INP0403, RCZ12, RCZ20^#^	WrbA*, Tpx*, FolX*, Ler, EspD^#^	*E. coli*	(Tree et al., 2009; Wang et al., 2011; Zambelloni et al., 2017)
INP0031*, INP0341	Unknown; Tpx*, FolX*	*Pseudomonas*	(Wang et al., 2011; Uusitalo et al., 2017; Sharma et al., 2020)
INP0007, INP0010, INP0031*, INP0400, INP0401, INP0402, INP0403, INP0404, INP0405, INP0406	Unknown; WrbA*, Tpx*	*Salmonella*	(Hudson et al., 2007; Negrea et al., 2007; Veenendaal et al., 2009; Wang et al., 2011)
INP0031*, INP0341, INP0400^#,^INP0402^#^, INP0404^#^,NP0405^#^	Unknown; WrbA*, Tpx*, FolX*, needle assembly^#^	*Shigella*	(Veenendaal et al., 2009; Wang et al., 2011)
Compound 4, INP0007, INP0010, INP0031*	Unknown, WrbA*, Tpx*	*Yersinia*	(Kauppi et al., 2003; Nordfelth et al., 2005; Wang et al., 2011)
B8I-1, -2, -3, -4, -5	VirB8	*Brucella*	(Paschos et al., 2011; Smith et al., 2012)

* and # = indicate connections between compounds and targets listed in the respective row

SAHs and their derivatives were first described as inhibitors of the *Yersinia* T3SS by Kauppi et al. (2003). The authors used a transcriptional fusion of the *yopE* gene (encoding the effector protein YopE) and luciferase and identified 30 molecules that significantly reduced luciferase expression. Compounds 2 to 4 decreased luciferase activity without or with only minor effects on bacterial growth. Furthermore, when grown in medium containing these compounds, Yop secretion was decreased in a dose-dependent manner. While being the most potent, Compound 2, an SAH, also affected bacterial motility. Compound 2, later renamed INP0007, was further investigated and shown to specifically block Yop secretion and translocation by *Yersinia* without adverse effects on bacterial or cell viability (Kauppi et al., 2003; Nordfelth et al., 2005).

Hudson et al. assessed the SAHs INP0007 and INP0403 as inhibitors of the *Salmonella* T3SS-1 (SPI-1). Both compounds inhibited effector secretion and SPI-1-mediated hemolysis in a dose-dependent manner. *In vitro* invasion assays with pre-incubated bacteria further showed a significant decrease in the number of intracellular bacteria in the presence of INP0007 (Hudson et al., 2007). Moreover, Negrea et al. tested nine SAHs (INP0007, INP0010, INP0400, INP0401, INP0402, INP0403, INP0404, INP0405, and INP0406) in *Salmonella* infections. All compounds reduced SPI-1-mediated invasion and effector secretion, and INP0400 and INP0007 also inhibited intracellular replication. All compounds, except INP0010, also inhibited the secretion of a SipB-β-lactamase fusion protein. In INP0010-treated cells, the fusion protein was expressed but not secreted. Furthermore, transcriptional reporter fusions suggested that this compound interfered with the transcription of SPI-1. INP0404 or INP0405 were shown to affect *Salmonella* motility (Negrea et al., 2007). Interestingly, INP0403 was later shown to reduce the transcription of genes involved in T3-secretion and iron acquisition and that its inhibitory activity was reversed when iron was supplied exogenously (Layton et al., 2010).

As the T3SSs of *Yersinia* and *Chlamydia* are highly conserved (Hsia et al., 1997), Wolf et al. investigated a potential role for INP0007 in inhibiting the T3SS of obligate intracellular *Chlamydia* (Wolf et al., 2006). Here, treatment with INP0007 inhibited *C. trachomatis* development in a dose-dependent manner, while bacterial viability was unaffected. Moreover, the bacteria remained metabolically active but could not divide, and their differentiation from reticulate to elementary bodies was inhibited. Addition of the inhibitor after infection blocked the conversion from established reticulate bodies back to elementary bodies in *in vitro *infection experiments. These effects were reversible when the inhibitor was removed (Nordfelth et al., 2005).

Additional well-characterized SAHs targeting *Chlamydia* are INP0010, INP0400, and INP0341. They were demonstrated to inhibit the *C. pneumoniae* developmental cycle (INP0010) and intracellular replication of *C. pneumoniae* and *C. trachomatis in vitro* (Muschiol et al., 2006; Bailey et al., 2007; Slepenkin et al., 2007). Treating epithelial cells with INP0010 significantly reduced intracellular levels of the effector proteins IncB and IncC. Transcription of *incB* was increased upon treatment, while *incC* transcription remained unaltered, indicating that INP0010 affected effector translocation but not gene expression (Bailey et al., 2007). While treatment with INP0400 at the time of infection partially blocked the entry of elementary *C. trachomatis* bodies into host cells, treatment with INP0400 during the mid-cycle reduced translocation of the effector protein IncA (Muschiol et al., 2006). INP0341, on the other hand, completely inhibited the expression of the T3SS chaperone LcrH-1 and the structural protein CopB in *C. trachomatis* during the mid- and late developmental stages (Slepenkin et al., 2003; Slepenkin et al., 2007). Consequently, downregulation of *incA* but not *incC* and *incG* could be observed. It was suggested that INP0341 works by sequestering iron within the host cell, inhibiting *Chlamydia* development within the host (Slepenkin et al., 2007). Further *in vitro* studies showed that this inhibitor affected neither essential members of the vaginal microbiome, nor the integrity of the vaginal mucosa, and showed only mild cytotoxicity. It was also observed that it could protect from vaginal *Chlamydia* infection in a mouse model (Slepenkin et al., 2011; Pedersen et al., 2014). After the initial *in vivo* experiments, the inhibitor was improved and formulated as a vaginal gel suitable for use in humans (Pedersen et al., 2014). Ur-Rehman et al. later investigated a library of SAHs for their effect on *C. pneumoniae* and *C. trachomatis* (Ur-Rehman et al., 2012) and chose ME0177 and ME0192 for further investigation due to their low toxicity. ME0192, the more potent compound, showed potent activity against intracellular chlamydial growth *in vitro*. However, its short half-life (< 1h) limits its application to topical treatment. Promisingly, the inhibitory effects of ME0192 were unaffected in the presence of vaginal simulant fluid or healthy vaginal microbiota. Hence, the effect of ME0192 was subsequently assessed *in vivo* by topical application before, during, and five days after *C. trachomatis* infection. Here, a significant reduction in the number of colonized mice and bacterial burden could be observed. ME0177, on the other hand, had a concentration-dependent effect *in vivo*, which lasted even after its removal, making it a promising compound for systemic treatment (Ur-Rehman et al., 2012).

INP0341 also inhibited the expression and secretion of the *Pseudomonas* exotoxin ExoS and *P. aeruginosa*-mediated cytotoxicity *in vitro*. In *in vivo* infection with *P. aeruginosa*, topical treatment of infected mice could delay mortality although it was unable to prevent the systemic spread of infection (Uusitalo et al., 2017). Subsequent research investigated its inhibitory effect on *P. aeruginosa* infections of human corneal epithelial cells and in a murine keratitis model (Sharma et al., 2020). These studies confirmed that INP0341 inhibited *P. aeruginosa*-mediated cytotoxicity without affecting bacterial viability. Additionally, *in vivo* experiments showed that topical application of INP0341 to corneal scratches could inhibit bacterial growth and facilitate bacterial clearance (Sharma et al., 2020).

The SAHs INP0400, INP0402, INP0404, and INP0405 were also investigated in *Shigella* infections, where they were shown to inhibit T3SS-mediated effector secretion. Moreover, the ability of *Shigella* to invade epithelial cells and cause apoptosis in macrophages was impaired. The most substantial effect could be observed when the compounds were added to the bacterial growth cultures. Electron microscopy analysis of inhibitor-treated bacteria demonstrated that these compounds interfered with the assembly of the *Shigella* T3SS needle (Veenendaal et al., 2009).

Tree et al. on the other hand discovered that the SAHs INP0010, INP0031, INP0401, and INP0403 did not affect the T3SS of enterohemorrhagic *E. coli* (EHEC) directly but acted *via* regulators, such as the locus of enterocyte effacement (LEE) regulator *ler* (Tree et al., 2009). For the most part, the mechanisms of action of the SAH inhibitors remain unclear. Later, new classes of SAHs were created by Zambelloni et al., using INP0031 as a scaffold. The authors developed eight novel compounds by incorporating the imine moiety into two different hydrazine-containing heterocycles. Two of these, RCZ12 and RCZ20, displayed potent EHEC T3SS inhibition. *In vitro* pull-down assays identified the coiled-coil domain 1 of the effector protein and translocon component EspD as the binding site of these inhibitors (Zambelloni et al., 2017).

The suppression of T3SSs of different pathogens by SAHs suggests that they may affect a conserved factor, with direct effects on T3S, the T3SS, or regulatory proteins being discussed (Tree et al., 2009; Veenendaal et al., 2009; Wang et al., 2011). For INP0031, Wang et al. identified three potential target proteins, WrbA, Tpx, and FolX, which they confirmed in different bacterial pathogens, including *E. coli, P. aeruginosa* and *Shigella*. All of these proteins are known to contribute to the expression of virulence factors, including T3SSs and flagella (Wang et al., 2011) and Tpx and WrbA have also been attributed an important role in motility activation (Nachin et al., 2005). SAHs may, therefore, increase the activity of Tpx and WrbA and enhance their repressive effects on the T3SS. As the tested inhibitors did not affect expression levels of Tpx and WrbA, the authors proposed a direct interaction of the inhibitor with these proteins, resulting in their activation or stabilization. SAHs may further bind to several proteins important for cellular stress responses, which, in turn, impair the transcription, expression, and assembly of the T3SS (Wang et al., 2011).

B8I-1 to -5 are salicylidene acylhydrazides with structural similarities to broad-spectrum inhibitors of T3SSs (Smith et al., 2012). Interestingly, they were identified to target VirB8 of the Vir T4SS of *Brucella abortus* and did not show broad-spectrum activity against other types of T4SSs, suggesting high target specificity (Paschos et al., 2011). B8I-2 inhibited T4SS assembly by binding within a surface groove near the dimerization site in VirB8, thus preventing VirB8 dimerization by inhibiting conformational changes. Moreover, it reduced the levels of several VirB proteins, suggesting that inhibitor binding destabilized the VirB8 protein (Smith et al., 2012).

## Other chemical secretion system inhibitors

Many high-throughput screens aimed at identifying secretion system inhibitors have used commercially-available chemical compound libraries. Some of these libraries also included well-characterized pharmacological substances. Various chemical substances have been identified as secretion system inhibitors in this way (**Table 2**).

**Table 2:** Other chemical secretion system inhibitors.

Compound name(s)	Molecular target	Effective against	Reference
1G2	Cagα	*Helicobacter*	(Arya et al., 2019)
2834, 3624, 9652	ATPase (Spa47)	*Shigella*	(Swietnicki et al., 2011; Case et al., 2018)
7812, 7832, 7086	ATPase (YscN)*, Unknown	Yersinia* *Burkholderia*	(Swietnicki et al., 2011)
8-amino imidazo[1,2-α]pyrazine	Cagα	*Helicobacter*	(Sayer et al., 2014)
C1, C3, C4, C5, C7	DotL, DotB	*Legionella* *Chlamydia*	(Cheng et al., 2022)
C10, KSK85	Unknown	*Helicobacter* *Agrobacterium*	(Shaffer et al., 2016)
C15, C19, C20*, C22*, C24*, C38*	Unknown	*Yersinia* *Pseudomonas	(Harmon et al., 2010)
CHIR-1, -2, -3	Unknown	*Helicobacter*	(Hilleringmann et al., 2006)
Cisplatin, Carboplatin	Unknown	*Helicobacter*	(Lettl et al., 2020)
Compound 1 Compound 2, Compound 3, Compound 4	Unknown	*Yersinia*	(Pan et al., 2009)
Compound D, Compound 2	Yop translocation	*Yersinia* *Pseudomonas*	(Pan et al., 2009; Jessen et al., 2014)
Cyclic peptomers (EpD-3´N, EpD-1,2´N, EpD-1,3´N, EpD-1,2,3´N, EpD-1,2,4´N and 4EpDN)	Needle assembly	*Yersinia* *Pseudomonas* *Salmonella* *Chlamydia*	(Lam et al., 2017; Lam et al., 2021)
Dey1, Dey 2, Dey 3, Dey 4	IpaD	*Shigella*	(Dey et al., 2017; Hotinger et al., 2021)
Fluorothiazinon	Unknown	*Salmonella* *Pseudomonas* *Chlamydia*	(Zigangirova et al., 2012; Koroleva et al., 2015; Nesterenko et al., 2016; Sheremet et al., 2018; Zigangirova et al., 2021)
S3, S4, S6	Unknown	*E. coli*	(Mühlen et al., 2021)
Thiazolidinone	Needle assembly, suggested: secretin	*Salmonella* *Yersinia* *Francisella*	(Felise et al., 2008)
W1227933, W1774182	ATPase (SctN)	*Chlamydia*	(Grishin et al., 2018)

* = indicates a connection between compounds or targets and pathogens listed in the respective row

### Chemical compounds interfering with T3SSs

Felise et al. screened for small molecules inhibiting the *Salmonella* T3SS using different effector proteins fused to a phospholipase. In this screen, the authors obtained seven hits that reduced the phospholipase activity and secretion of effector proteins. However, only thiazolidinone did not affect the production of transcriptional regulators of the T3SS machinery. This compound inhibited the formation of needle complexes and thus blocked the T3SSs in *Yersinia* and *Francisella*. Additionally, it blocked the T2SS, but not the function of flagella in *Francisella*, suggesting the targeting of secretin, the only protein homolog shared between T2- and T3SSs (Felise et al., 2008).

In an early study, Pan et al. screened a chemical compound library for substances that reversed bacterial growth inhibition, which occurs in *Y. pestis* under T3 secretion conditions. Four inhibitory compounds were identified, designated Compound 1-4. All of these compounds showed a concentration-dependent inhibition of Yop effector secretion. Interestingly, Compounds 1 and 3 inhibited the secretion of Yops to varying degrees. Compounds 1, 3, and 4 only inhibited T3S-mediated cytotoxicity, while Compound 3 also inhibited the translocation of a YopE-β-lactamase fusion protein. Compounds 1 and 3 further inhibited T3-mediated secretion of the effector protein Tir from enteropathogenic *E. coli* (EPEC) (Pan et al., 2009). Jessen et al. tested Compound 2 and its isomer Compound D for their effect on *Y. pestis* (Jessen et al., 2014). They confirmed that Compound D also inhibited Yop secretion and additionally observed the inhibition of effector protein secretion by *P. aeruginosa* and *Y. pseudotuberculosis.* While the mechanisms remain unknown, it was suggested that Compound D inhibits Yop translocation *via* interaction with the translocon component YopD. Compound 2 appears to employ a different mechanism as, unlike Compound D, it was also active in the presence of the *Yersinia* Yop secretion inhibitor calcium. Unfortunately, both compounds were toxic to eukaryotic cells, and their modification will be necessary to improve safety (Jessen et al., 2014).

Six additional chemical compounds were identified, which block the translocation of a *Y. pseudotuberculosis* YopB-β-lactamase fusion protein into eukaryotic cells. In the study, C15, C19, C20, C22, C24, and C38 inhibited the YopB translocation without affecting the expression of T3SS components or effector proteins, formation of the T3SS, or the secretion of effectors. C20 was further shown to reduce the binding of *Yersinia* to host cells. Interestingly, all compounds except C15 and C19 also inhibited ExoS secretion from *P. aeruginosa*, as shown by the reduction of ExoS-mediated cell death. Therefore, the molecular targets of these compounds appear to be conserved between *Y. pseudotuberculosis* and *P. aeruginosa* (Harmon et al., 2010).

Zigangirova et al. developed a new small-molecule inhibitor, Fluorothiazinon (FT, previously called CL-55) (Zigangirova et al., 2012). FT was one of the first T3SS inhibitors which showed potential for therapeutic treatment of *Salmonella* infections (Nesterenko et al., 2016; Zigangirova et al., 2021). It was characterized by low toxicity, high stability, and therapeutic efficacy in animal models (Sheremet et al., 2018). FT inhibited the secretion of early *Salmonella* SPI-1 effectors without affecting intracellular bacterial growth (Zigangirova et al., 2021), while suppressing bacterial growth within macrophages. This suggested that FT inhibited not only SPI-1 but also *Salmonella* T3SS SPI-2, which is essential for intracellular survival and systemic spread (Zigangirova et al., 2021). In a mouse model of acute *Salmonella* infection, treatment with FT decreased the bacterial load and increased survival rates (Nesterenko et al., 2016; Sheremet et al., 2018). Further investigation in a murine model of systemic *Salmonella* infection showed that FT could suppress systemic spread when used as either therapeutic (seven days after infection) or preventative treatment (four days before infection) (Zigangirova et al., 2021). In a *P. aeruginosa in vivo* airway infection model, FT inhibited the secretion of the effector proteins ExoT and ExoY in a dose-dependent manner. Moreover, it was shown that the treatment could reduce tissue damage and prevent systemic spread and mortality (Sheremet et al., 2018). A phase II trial is currently underway, which includes FT as an active ingredient in the treatment of patients with urinary infections caused by *P. aeruginosa* (Zigangirova et al., 2021). FT was further shown to block the T3SS-mediated virulence of *C. trachomatis in vitro*. Upon treatment, the effector protein IncA was absent from chlamydial inclusions, whereas its transcription in bacteria was unimpaired, suggesting that FT affects the T3SS and not bacterial metabolism (Zigangirova et al., 2012). In a subsequent study, the inhibitory effect of FT was further studied in *C. trachomatis*-infected mice. Here, the number of bacteria in vaginal excretions was reduced upon infection, and a decrease in chlamydial DNA was observed in ovaries and uteri (Koroleva et al., 2015).

Schwochert et al. designed a library of synthetic cyclic peptomers (Schwochert et al., 2016) inspired by phepropeptin D produced by *Streptomyces* (Sekizawa et al., 2001), which Lam et al. used to screen for inhibitors of NF-κB activation in response to *Yersinia* infection. They showed that EpD-3’N, EpD-1,2N, EpD-1,3’N, EpD-1,2,3’N, and EpD-1,2,4’N strongly inhibited effector secretion by *Yersinia* and *P. aeruginosa* without affecting bacterial growth and with only low cytotoxicity in mammalian cells (Lam et al., 2017). The authors then developed isomers based on the cyclic peptomer EpD-1,2N. They observed that 4EpDN was more potent than its parental compound and that it inhibited the T3SSs of both, *Yersinia* and *S*. Typhimurium without affecting the transcription of T3SS genes. In addition, 4EpDN reduced the number of T3SS needles on the surface of *Y. pseudotuberculosis*, suggesting that it may specifically prevent needle assembly in a variety of pathogens (Lam et al., 2021). Because the compound reduced the localization of the inner ring protein YscD to the *Yersinia* inner cell membrane, it has been suggested that 4EpDN may interact with the lumen of the T3SS needle, affecting the secretion of large cargo proteins more than that of small cargo. Alternatively, it may disrupt the assembly of the T3SS needle subunit, thereby resulting in non-functional needles and impairing effector translocation (Lam et al., 2021).

Other small molecules inhibiting needle formation were identified by Dey et al., who performed a structure-based compound screen to identify molecules binding to the effector protein IpaD of *Shigella* (Dey et al., 2017). The authors found four small molecules with scaffolds based on quinoline, pyrrolidine aniline, hydroxyindole, and morpholinoaniline, later named Dey 1-4, respectively (Hotinger et al., 2021). After binding of the compounds to IpaD, conformational changes were induced, inhibiting translocon assembly by a yet unknown mechanism (Dey et al., 2017).

The effector protein Tir is essential for intimate attachment to enterocytes by EPEC and EHEC. Mühlen et al. recently described the inhibitory substances S3, S4, and S6, which reduced the translocation of a Tir-β−lactamase fusion protein, as well as Tir-mediated attaching and effacing (AE) lesion formation and effector-dependent cell detachment. None of the compounds affected the expression of T3SS components EspA, EspB, or EspD or that of the effector protein Tir. The three identified compounds belong to different chemical classes and varied in their ability to inhibit T3SS-mediated hemolysis, suggesting that they have different modes of action. As neither of the compounds induced the expression of Shiga toxin in a Shiga toxin reporter strain, they may be promising candidates for treating EHEC infections if developed further (Mühlen et al., 2021).

### Chemical inhibitors targeting T4SSs

While T3SSs have been the focus of many inhibitor screens, few studies have aimed to identify substances, which can interfere with T4SS-mediated virulence. In 2016, Shaffer et al. identified two closely related small molecules, called C10 and KSK85, which impaired delivery of the *H. pylori* T4-effector CagA and peptidoglycan into host cells. KSK85 inhibited the formation of Cag secretion system-associated pili, while C10 impaired protein transport. Neither inhibitor showed any adverse effects on the viability of gastric epithelial cells or bacteria (Shaffer et al., 2016). A distinctive feature of these compounds was their activity against other T4SSs, such as the Vir T4SS of *A. tumefaciens* (Shaffer et al., 2016).

Lettl et al. screened a library of pharmaceutically-active compounds for their ability to inhibit CagA-β-lactamase translocation by *H. pylori*. They identified two potent platinum coordination complexes, cisplatin and carboplatin, which are known for their anti-tumorigenic functions (Jamieson and Lippard, 1999). These compounds were further investigated by measuring CagA translocation into eukaryotic cells upon *H. pylori* infection. Both compounds reduced translocation and, hence, the intracellular phosphorylation of CagA. The viability of *H. pylori* was not affected. Additionally, the levels of IL-8, usually increased upon infection, were reduced by cisplatin treatment in a dose-dependent manner as was the bacterial adherence to host cells in the presence of cisplatin. Interestingly, once *H. pylori* infection was established, the adherence could not be resolved. However, the translocation of CagA was still reduced, suggesting that cisplatin can rapidly interfere with and inhibit effector translocation (Lettl et al., 2020).

Using a high-throughput screen to identify compounds that interfere with the translocation of effector proteins from *Legionella pneumophila* into macrophages, Cheng et al. identified five compounds (C1, C3, C4, C5, and C7), which showed high efficiency in different assays that require a functional T4SS. These included β-lactamase-LidA translocation and intracellular replication in macrophages. While C4 was shown to reduce the levels of LidA-reporter protein translocation by *L. pneumophila* but not by *Coxiella burnetii*, compounds C1, C3, C5, and C7 reduced effector translocation from both bacteria. Additionally, none of the compounds showed any adverse effects on the growth of *L. pneumophila* or *C. burnetii* in culture. As the five compounds vary in molecular structure and chemical properties, the authors suggested they may have diverse targets (Cheng et al., 2022).

### Chemical inhibitors of secretion system-powering ATPases

Secretion system-associated ATPases are required for powering the secretion of needle/pilus components and effector proteins into host cells. Swietnicki et al. used a structure-based screen for chemical compounds which could bind the *Yersinia* T3SS ATPase YscN. They identified the compounds 7812, 7832, and 7086. These compounds were confirmed to potently inhibit YscN in an *in vitro* ATPase assay and subsequently abolished the secretion of YopE into host cells upon infection. Furthermore, these compounds also inhibited the ATPase BsaS of the *Burkholderia mallei* T3SS, which shows sequence homology to YscN. Because the identified compounds were not or only slightly toxic to mammalian cells, they do not appear to interfere with the function of eukaryotic ATPases (Swietnicki et al., 2011).

In the *H. pylori* Cag T4SSs, the ATPase Cagα powers complex assembly, DNA unwinding and translocation (Hilleringmann et al., 2006). Hilleringmann et al. identified three inhibitors of Cagα ATPase activity, CHIR-1, -2, and -3. Due to its potency, CHIR-1 was used for further characterization. It inhibited CagA secretion by *H. pylori* in a dose-dependent manner, completely blocking its translocation after pre-incubation, suggesting it may interfere with the early stages of T4SS assembly. Additionally, pre-incubation of *H. pylori* with CHIR-1 before intragastric infection of mice decreased the number of infected animals as well as their colonization (Hilleringmann et al., 2006).

Sayer et al. later tested 8-amino imidazo[1,2-α]pyrazine derivates as inhibitors of Cagα. To characterize the inhibitory effects of these compounds, they purified Cagα from *E. coli* and tested it in an *in vitro* ATPase assay. The authors observed a partial inhibition of ATPase activity in the presence of Compounds 11 and 32. A prediction of the docking site of Compound 11 suggested that the inhibitor binds within the catalytic site (Sayer et al., 2014). However, the solubility of the compound was low and needed modification. To improve their selectivity for VirB11 ATPases, the authors later added a peptide sequence to the previously identified substances in an attempt to disrupt peptide-peptide interactions. Here, the inhibitory effect was increased in *in vitro* ATPase assays when the compound was linked to a PEG-carbamate linker (Sayer et al., 2021).

Further work focusing on Cagα used a fragment-based screening approach for molecules that bound to or stabilized the ATPase in an *in vitro* ATPase assay. The authors identified four fragments, of which 1G2 was the most potent. The binding of 1G2 to the ATPase led to alterations in Cagα conformation and appeared to interfere with Cagα hexamer formation. The inhibition appears to be partial since treatment of bacteria with 1G2 reduced CagA-induced IL-8 production in cells upon infection, but intracellular phosphorylation of CagA within the host cells remained unaffected (Arya et al., 2019).

Also using *in silico* docking simulations, Case et al. identified three inhibitors of the T3SS ATPase Spa47 of *Shigella*. These compounds (2834, 3624, and 9652) showed decreased ATPase activity in ATPase assays and suppressed effector protein secretion without significant effects on bacterial growth or cell viability. The study further indicated that the inhibitors did not act by disrupting Spa47 oligomers or by preventing T3SS formation (Case et al., 2018).

SctN is the ATPase powering the T3SS of *C. trachomatis* (Beeckman et al., 2008). Using structure-based virtual screening against modeled SctN, two compounds, W1227933 and W1774182, were identified, which bind to SctN, blocking the translocation of the effector protein IncA (Grishin et al., 2018). A significant decrease was observed in the number of intracellular *C. trachomatis* inclusions when cells were treated with W1227933 and W1774182 at the time of infection. Intracellular chlamydial survival was completely abolished at high concentrations. Both compounds showed low to medium concentration-dependent cytotoxicity in eukaryotic cells. As the exact structure of SctN is still unknown, Grishin et al. used homologous ATPases, such as the *E. coli* T3SS ATPase EscN and the *Salmonella enterica* flagellar ATPase FliI for modeling, suggesting that these ATPases may also be targeted (Grishin et al., 2018).

## Natural compounds inhibiting virulence-associated secretion systems

While most antibiotics were derived from natural sources, most screens for secretion system inhibitors have used commercially-available chemical compound libraries. Lately, however, there has been a shift back to assessing the potential of natural compounds in targeting bacterial infections and a number of promising substances have been identified (**Table 3**).

**Table 3:** Natural secretion system inhibitors.

Compound name(s)	Molecular target	Source	Effective against	Inhibitory concentration	Reference
(-)-Hopeaphenol	Unknown	Dipterocarpaceae *e.g., Shorea ovalis*	*Yersinia* *Pseudomonas* *Chlamydia*	IC_50_: 6.6 and 3.3 µM	(Zetterström et al., 2013)
Aurodox	*ler*	*Streptomyces goldiniensis*	*E. coli* *C. rodentium*	1.5 µg/mL	(Kimura et al., 2011) (McHugh et al., 2019; McHugh et al., 2022)
Biparatopic nanobodies 13F07-5H01, 13F07-14E10 and 1E11-5H01	PcrV	Camelid Ig structure	*Pseudomonas*	IC_50_: 10^-6^ - 4x10^-9^ M	(De Tavernier et al., 2016)
Caminoside A	Unknown	*Caminus sphaeroconia*	*E. coli*	IC_50_: 20 µM	(Linington et al., 2002)
Cinnamaldehyde	Unknown	*Cinnamomum* sp.	*Salmonella*	–	(Liu et al., 2019)
Cytosporone B	Unknown	*Sordariomycetes* sp.	*Salmonella*	IC_50_: 6.25 µM	(Li et al., 2013)
(-)-Epigallocatechin gallate (EGCG)	Unknown	Green tea	*E. coli* *Salmonella*	4 – 500 µg/mLIC_50_: 2.15 µM	(Nakasone et al., 2017; Tsou et al., 2017)
Fusaric acid derivates SL-8 and SL-19	Unknown	*Fusarium heterosporium*	*Salmonella*	IC_50_: 53.5 µM	(Li et al., 2014; Song et al., 2021)
Guadinomines A, B, C1, C2 and D	Unknown	*Streptomyces* sp.	*E. coli*	IC_50_: 0.03 µg/mL (Guadinomine A) IC_50_: 0.007 µg/mL (Guadinomine B) IC50: 8.5 µg/mL (Guadinomine D)	(Iwatsuki et al., 2008a)
Lactoferrin	Unknown	mammals 0.125 mM 10 mg/mL	*E. coli* *Shigella*	0.125 mM10 mg/mL	(Gomez et al., 2003; Ochoa et al., 2003; Ochoa and Clearly, 2004; Ochoa et al., 2004)
Myricetin	Unknown	flavonoid, common dietary source	*Salmonella*	–	(Lv et al., 2021)
Obovatol	Unknown	*Magnolia obovate*	*Salmonella*	IC_50_: 19.8 µM	(Choi et al., 2017)
Paeonol	Unknown	Peonies *e.g*., *Paeonia suffruticosa*, *Arisaema erubescens*, *Dioscorea japonica*	*Salmonella*	0.9 mmol/L0.048 - 0.76 mM	(Lv et al., 2020)
Piericidin A1 and Mer-A 2026B	Unknown; Suggested role in needle assembly	*Streptomyces mobaraensis*	*Yersinia*	71 µM	(Duncan et al., 2014; Morgan et al., 2017)
Prenylated flavonoids (Licoflavonol)	Unknown	*Macaranga* sp.	*Salmonella*	0-50 µM	(Guo et al., 2016)
Sanguinarine chloride	Unknown	*Sanguinarine canadensis*	*Salmonella*	5 µM	(Zhang et al., 2018b)
Syringaldehyde	Unknown	Spruce, Maple trees	*Salmonella*	0.045 – 0.72 mM	(Lv et al., 2019)
Tanshinones 12(6,4) and 12(4,6)	Unknown	*Salvia miltiorrhiza*	*Pseudomonas*	IC_50_: 15 µM (12(6,4)), 9 µM (12(4,6))	(Ngo et al., 2019)
Thymol	Unknown	*Thymus* ssp.	*Salmonella*	IC_50_: 0.05 mM	(Zhang et al., 2018a; Qi et al., 2020; Zhang et al., 2021)

### Natural inhibitors isolated from animal sources

The glycolipid caminoside A was found in extracts of the marine sponge *Caminus sphaeroconia* and was the first T3SS inhibitor ever described. It decreased the secretion of the EPEC translocon component EspB into the supernatant without affecting bacterial growth or the secretion of other proteins (Linington et al., 2002).

One of the best-studied compounds is lactoferrin, which is widely represented in various body fluids such as milk, tears, and saliva and, thus, cytotoxicity is highly unlikely. Besides its bacteriostatic effect due to iron sequestration, it also decreased the virulence of several pathogens, including *Salmonella*, *Shigella*, and *E. coli*, by targeting their T3SS (Ochoa et al., 2003; Ochoa et al., 2004; Bessler et al., 2006; Ochoa and Cleary, 2009; Mosquito et al., 2010). Lactoferrin induced the degradation of the *Shigella* translocon proteins IpaB and IpaC (Gomez et al., 2003) and the *E. coli* tip and translocon proteins EspA, EspB, and EspD (Ochoa et al., 2004). Interestingly, it functions *via* two distinct mechanisms. By binding LPS, protein-protein interactions were disrupted, resulting in the instability of virulence proteins. Additionally, it directly degraded T3SS components through its intrinsic serine protease activity.

In addition, antibodies derived from camelids, so-called nanobodies, can also be used to inhibit the T3SS. De Tavernier et al. screened a large library of bivalent and biparatopic nanobodies for the functional inhibition of PcrV, which is an integral part of the *P. aeruginosa* T3SS, as it forms the needle tip (Goure et al., 2004; Goure et al., 2005; Sato and Frank, 2011). This screen yielded a group of nanobodies (13F07-5H01, 13F07-14E10, and 1E11-5H01) which inhibited *P. aeruginosa*-mediated cell death. *In vivo* mouse models of an acute, lethal *P. aeruginosa* lung infection showed 100% survival when the nanobodies were used as prophylactic treatment. 13F07-5H01 significantly improved the survival of infected mice under therapeutic conditions, when treatment started as late as 18 hours post-infection (De Tavernier et al., 2016).

### Secretions system inhibitors derived from bacteria and fungi

In 2008, Iwatsuki et al. identified six inhibitory compounds produced in *Streptomyces* sp.: guadinomines A, B, C1, C2, and D, and guadinomic acid. All of these compounds exhibited dose-dependent inhibition of T3SS-mediated hemolysis by EPEC without any cytotoxic or antimicrobial effects (Iwatsuki et al., 2008a; Iwatsuki et al., 2008b).

Another T3SS inhibitor isolated from *Streptomyces* is the kirromycin derivative aurodox (Kimura et al., 2011). This linear polyketide compound is highly similar to kirromycin, from which it differs only in the methylation of its pyridone moiety (McHugh et al., 2022). Aurodox was first identified in a screen for inhibitors of T3SS-mediated hemolysis in EPEC and was subsequently shown to decrease the secretion of effector proteins EspB, EspF, and Map without affecting bacterial growth. Furthermore, aurodox protected mice from succumbing to a lethal dose of *Citrobacter rodentium*, the mouse homolog of EPEC (Kimura et al., 2011). Later, aurodox was shown to inhibit the T3SS and, hence, virulence in EHEC and EPEC by downregulating the transcription of the virulence master regulator, *ler*. As aurodox did not induce the expression of Shiga toxin in EHEC, the compound may be a promising treatment option for EHEC infections (McHugh et al., 2019).

Another well-studied natural compound is cytosporone B (Csn-B), which is produced by fungi of the class *Sordariomycetes*. Li et al. screened a library of cytosporone B analogs and identified eight compounds (Csn-B and seven analogs) as inhibitors of *S.* Typhimurium SPI-1. Four compounds inhibited the secretion of SPI-1 effector proteins, and three of these also interfered with *Salmonella* invasion into eukaryotic cells (Li et al., 2013). Csn-B was the most potent compound, inhibiting effector protein secretion in a dose-dependent manner without affecting the flagellar T3SS or adverse effects on bacterial cells. Interestingly, overexpression of the T3SS regulator HilD could counteract Csn-B inhibition, suggesting that the compound interferes with the HilD regulatory cascade. Indeed, Csn-B upregulated the transcription of *hha* and *hns* encoding Hha and H-NS, which bind to promoters of SPI-1 regulatory genes, including *hilD* to repress their transcription (Ellermeier et al., 2005; Olekhnovich and Kadner, 2007; Li et al., 2013).

In 2014, Duncan et al. reported the discovery and initial characterization of two new *Streptomyces*-derived T3SS-inhibitors, Piericidin A1 and its derivative Mer-A 2026B. In their screen, the authors showed that both compounds prevented T3SS-dependent activation of NF-κB by *Y. pseudotuberculosis* effector proteins. Both Piericidin A1 and Mer-A 2026B inhibited the translocation of YopM into eukaryotic cells in a dose-dependent manner. Furthermore, neither compound interfered with bacterial growth or showed toxicity when added to eukaryotic cells, suggesting inhibition of an early stage, such as T3SS needle assembly (Duncan et al., 2014) instead of blocking bacteria-host cell interactions. In a later study, Morgan et al. confirmed this hypothesis by observing a reduced number of needle complexes at the bacterial surface combined with a decreased T3SS formation and effector translocation in Piercidin A1-treated *Yersinia* (Morgan et al., 2017).

Li et al. initially identified the mycotoxin fusaric acid (FA) as an inhibitor of the *Salmonella* T3SS. FA inhibited the secretion of SPI-1 effectors and bacterial invasion into host cells without disrupting cell growth or cytotoxicity (Li et al., 2014). Later, Song et al. designed twenty-two diphenylsulfane derivatives based on FA. Among these, SL-8 and SL-19 possessed strong activities against the T3SS and bacterial invasion and showed an improved potency compared to FA. The inhibitory effect on SPI-1 was independent of regulatory genes and the assembly of the T3SS needle complex. Thus, the authors proposed that SL-8 and SL-19 influence the formation of the SicA/InvF-effector complex or other related proteins, which may act as classical transcriptional activators (Song et al., 2021).

### Plant-based pathoblockers

(-)-Hopeaphenol is a plant-derived inhibitor that targets the T3SSs of different pathogens, including *Y. pseudotuberculosis* and *P. aeruginosa* (Zetterström et al., 2013). It is highly efficient in blocking the expression of the *Yersinia* effector protein YopE. It further interfered with the translocation of ExoS from *P. aeruginosa* at higher concentrations. Moreover, (-)-hopeaphenol reduced entry and subsequent intracellular growth of *C. trachomatis*. Interestingly, *Y. pseudotuberculosis* grown in the presence of (-)-hopeaphenol did not assemble T3SSs when transferred to T3SS-inducing conditions, suggesting an irreversible inhibitory effect. It was suggested that (-)-hopeaphenol interacts with T3SS components at the bacterial surface, as its molecular weight and size imply a low membrane permeability (Zetterström et al., 2013). As (-)-hopeaphenol did not affect cell viability and growth, it may be a promising drug against multiple Gram-negative pathogens. Unfortunately, (-)-hopeaphenol is produced by plants of the *Anisoptera* spp. which are endangered, making the compound impossible to access and synthesize for further studies (International Union for Conservation of Nature (IUCN) Red List, 2019).

Tanshinones are natural herbal compounds isolated from the plant *Salvia miltiorrhiza*. The compounds were shown to block the interaction of the *P. aeruginosa* needle protein PscF with its chaperones PscE and PscG. Hence, they prevented needle formation and subsequent effector translocation of ExoS into infected macrophages. Additionally, a dose-dependent inhibition of intracellular bacterial survival was observed without cytotoxic effects for the host cell. Furthermore, mice were challenged with lethal doses of *P. aeruginosa* in a model of acute pneumonia to investigate the effect of tanshinones *in vivo*. There, the compounds reduced the secretion of bacterial virulence factors and prevented mice from dying when the inhibitors were applied at the time of infection and every 12 h afterwards (Quinaud et al., 2005; Abrusci et al., 2014; Feng et al., 2019). Another research group found two other tanshinone deviates, Clioquniol and 3-APPA1, by target-based screening, which the authors assembled into a series of larger hybrids. Here, two potent inhibitors were identified, 12(6,4) and 12(4,6). Like other tanshinones, they interfered with needle assembly by interacting with PscG and PscE. These compounds strongly inhibited the secretion of effector proteins ExoS and ExoT, while the secretion of PopD was dose-dependent. Both inhibitors were non-cytotoxic in eukaryotic cells and did not affect bacterial viability. *In vivo* experiments performed in *Galleria mellonella* showed that these tanshinone derivatives significantly improve larval survival upon *P. aeruginosa* infection (Ngo et al., 2019).

In 2016, Guo et al. tested twenty prenylated flavonoids (1-20), a unique class of naturally occurring flavonoids isolated from trees of the *Macaranga* species, for their inhibitory activity against effector secretion by SPI-1. Among the tested flavonoids, licoflavonol exhibited the most substantial inhibitory effect on the secretion of SPI-1-associated effector proteins from *Salmonella* Typhimurium. Furthermore, it did not affect bacterial growth or secretion of the flagellar protein FliC (Guo et al., 2016).

(-)-Epigallocatechin gallate (EGCG), is a catechin most abundant in green tea. After infection of EGCG-treated epithelial cells with EPEC or EHEC, the bacterial adherence to cells was significantly reduced (Nakasone et al., 2017). Similarly, upon *Salmonella* or *Yersinia* infection of EGCG-treated epithelial cells, invasion was inhibited without affecting bacterial growth (Tsou et al., 2017).

In 2017, obovatol, derived from the *Magnolia obovate* plant, was shown to potently inhibit the T3SS of *Salmonella* Typhimurium (Choi et al., 2017). It blocked bacterial motility, mRNA expression, and effector protein secretion without inhibiting bacterial growth. Furthermore, obovatol was able to block *Salmonella*-induced hemolysis (Choi et al., 2017).

Another plant-derived T3SS inhibitor is sanguinarine chloride, isolated from the bloodroot plant *Sanguinaria canadensis* (Babich et al., 1996). In the 1970s and 80s, it was studied as a promising gingivitis treatment due to its anti-inflammatory properties. However, its high cytotoxicity mainly limits its therapeutic use to chemotherapy (Mondal et al., 2019). Sanguinarine chloride potently inhibited SipA-β−lactamase fusion protein translocation into epithelial cells and reduced the invasion of *Salmonella*. Additionally, it reduced the expression of the effector proteins SipA and SipB. However, sanguinarine chloride decreased the level of HilA, suggesting inhibition of HilA-induced virulence genes, as overexpression of HilA could compensate for these inhibitory effects (Zhang et al., 2018b).

Cinnamaldehyde is the active compound of cinnamon oil which can be extracted from a plant of the *Cinnamomum* genus. It has been described to exhibit anti-bacterial, anti-inflammatory, and anti-oxidative functions (Zhu et al., 2017). The compound reduced the expression of *Salmonella* SPI-1 effector proteins by downregulating the transcription of virulence regulator genes, including *sipA* and *sipB* (Liu et al., 2019). Hence, it is unsurprising that cinnamaldehyde blocks the translocation of several SPI-1-associated effector proteins. Furthermore, cinnamaldehyde-treated mice infected with *S.* Typhimurium showed decreased colonization and increased intestinal barrier functions compared to untreated animals.

Syringaldehyde can be found in the wood of spruce and maple trees and also acted as an effective inhibitor of SPI-1. Treatment with syringaldehyde potently inhibited the expression of the effector proteins SipA, SipB, and SipC, blocking bacterial invasion and subsequent cellular damage during infection of epithelial cells with *Salmonella* Typhimurium without affecting bacterial growth. The inhibitory effect of syringaldehyde was also confirmed *in vivo*. Here, *S.* Typhimurium-infected mice showed reduced bacterial loads, cecal damage, and systemic inflammation, resulting in an overall decrease in mortality (Lv et al., 2019).

The essential oil thymol derives from plants belonging to the *Thymus* genus. Thymol inhibited *S. *Typhimurium invasion into mammalian cells and protected mice from infection (Zhang et al., 2018a). Proteomic analysis of thymol-treated cells revealed the differential regulation of 144 proteins involved in metabolism and cellular structure. This suggests an antimicrobial role of thymol in altering membrane permeability, antioxidant response, and virulence of *S.* Typhimurium (Qi et al., 2020). A follow-up study by Zhang et al. revealed that thymol directly interacted with SipA, inducing conformational changes in the protein, making SipA susceptible to degradation by the Lon protease (Zhang et al., 2021).

The phenolic compound paeonol, found in peonies, was recently identified as an effective *Salmonella* SPI-1 inhibitor. It blocked the translocation of SipA into host cells without affecting bacterial growth. Moreover, paeonol-treated mice infected with *S.* Typhimurium showed decreased invasion of the bacteria into tissues, hence reducing tissue damage (Lv et al., 2020). Mechanistic studies revealed the transcriptional downregulation of the SPI-1 regulator *hilA*, resulting in the inhibition of effector protein expression (Lv et al., 2020).

Lastly, myricetin, a flavonoid with antioxidant properties that can be found in vegetables, fruits, and red wine (Lv et al., 2021) was recently discovered to prevent *S.* Typhimurium invasion into host cells, decreasing cell damage. Moreover, it blocked the translocation of the effector proteins SipA and SipB. Further analysis showed that it interfered with the regulatory network of SPI-1-related genes, leading to a significant decrease in the expression of crucial effector proteins, ultimately inhibiting T3SS-mediated virulence. *In vivo*, it was shown that myricetin treatment prevented pathological damage and death in infected mice (Lv et al., 2021).

## Recent advances and the challenges for translation into the clinic

For over a decade, numerous high-throughput screens for pathoblockers have been conducted. In contrast to screening approaches for antibiotics, initial screens for secretion system inhibitors focused on commercially-available and sometimes overlapping chemical compound libraries (Duncan et al., 2012; Beckham and Roe, 2014; Boudaher and Shaffer, 2019; Hotinger et al., 2021). However, in recent years more and more natural compounds have been discovered which inhibit virulence-associated secretion systems in bacterial pathogens (Pendergrass and May, 2019). Unfortunately, the molecular targets of many pathoblockers identified over the years remain unknown, and no further research has been conducted to elucidate them. However, determining the targets of pathoblockers would enable their structural improvement (e.g., by chemical modification), potentially improving their efficacy or cell permeability and limiting cytotoxicity as well as potential cross-reactivity with eukaryotic proteins or processes (Miethke et al., 2021).

Several secretion system inhibitors have now been tested in mouse models of infection for their ability to inhibit virulence *in vivo* with encouraging results. However, the progression of promising compounds into clinical trials is hampered by difficulties in evaluating compound efficacy. While the therapeutic concentrations of antimicrobials targeting bacterial survival mechanisms are evaluated based on their minimal inhibitory concentration (MIC), this cannot be used as a guideline in the case of anti-infectives. Here, treatment does not lead to a measurable phenotype. Therefore, animal models are essential to determine the approximate therapeutic compound concentrations required for assessment in human trials.

Also, there is the question of bioavailability as well as the method of application. Topical treatments may be used for infections with *Chlamydia* or skin infections by *P. aeruginosa*. For other infections, oral administration is suggested for gastrointestinal infection with *H. pylori*, *S. enterica*, *Shigella* spp., and pathogenic *E. coli*, as well as enteropathogenic *Yersinia* species. For this, the compound has to be stable during its passage through the gastrointestinal tract and needs to be released at the site of infection. Pathoblockers targeting lung pathogens may need to be administered either as aerosols or by systemic application, posing yet other challenges.

Moreover, the potential of the different compounds as prophylactic versus therapeutic treatment must be considered. Most pathogens downregulate the expression of virulence genes including the energy-costly secretion systems after successful colonization of their target tissues (Heithoff et al., 1997; Heroven and Dersch, 2014; Connolly et al., 2018; Platenkamp and Mellies, 2018; Wang et al., 2019). *Salmonella* switches off the expression of SPI-1 upon invasion of host cells and, in turn, expresses SPI-2 once inside the host cell in a tightly regulated process to allow its survival and replication within the host (Lober et al., 2006; Singer et al., 2014; Lou et al., 2019). Yet other pathogens, such as *C. burnetii*, only express their secretion system once conditions inside host cells are favorable (Newton et al., 2020), escaping the recognition by secretion system inhibitors outside cells and limiting therapeutic treatment options.

## Conclusion

With AMR on the rise and the number of last-resort antibiotics dwindling by the day, the focus has to shift towards intervention therapies that render pathogens avirulent but do not affect their viability to circumvent resistance development. With expression, assembly, and effector translocation of secretion systems being a metabolically-costly endeavor for the bacteria, interference with this process should not cause the (rapid) development of resistance. Future studies will have to determine the function and targets of identified inhibitors to modify the structures to make them either more or less specific. As secretion systems are highly similar in structure, it has surprised many that most pathoblockers appear specific to the pathogen they were tested in and showed little to no overlap between species. This suggests that these inhibitors were not targeting the virulence-associated secretion systems but other proteins found in the pathogen, specific for regulating the expression/formation of secretion systems. Researchers have attempted to circumvent these issues by structure-guided inhibitor synthesis. However, this approach, just as those approaches trying to identify inhibitors using overexpressed secreted proteins, has the drawback of providing no evidence of whether the identified molecules are functional in a cell-based setting. Furthermore, depending on the proposed target, pathoblockers have to be cell-permeable to reach their targets if these are not exposed on the bacterial cell surface. Several obligate or facultative intracellular bacteria including *Coxiella*, *Shigella*, and *Chlamydia* will also require the inhibitory compounds to be membrane-permeable in order to reach the bacteria and/or their potential targets. Membrane crossing compounds, do, however, pose the risk of affecting other intracellular host cell processes. Furthermore, due to the evolutionary relationship between bacterial flagella and T3SSs, compounds targeting the T3SS may always also affect bacterial motility. While in itself not affecting bacterial viability and, therefore, not inducing selective pressure, targeting flagella and hence motility will also affect motile members of the intestinal microbiota, which may result in the loss of these bacteria and may, in turn, suppress colonization resistance.

There is still a long road ahead, but the drastic increase in AMR and the limited number of approved antibiotics over the last decade call for more intense efforts to identify novel lead compounds available for anti-virulence approaches and to further develop these compounds in order to pave their way into the clinic.

## Author contributions

All authors have made a substantial, direct, and intellectual contribution to this work and approved it for publication. All authors contributed to the article and approved the submitted version.
